# TNFRSF9 Suppressed the Progression of Breast Cancer via the p38MAPK/PAX6 Signaling Pathway

**DOI:** 10.1155/2022/8549781

**Published:** 2022-06-28

**Authors:** Xiaorong Liu, Yehui Zhou, Chenglin Qin, Xun Zhu

**Affiliations:** ^1^Department of General Surgery, The Second Affiliated Hospital of Soochow University, Suzhou, Jiangsu 215004, China; ^2^Department of General Surgery, The Second Affiliated Hospital of Jiaxing University, No. 1518 North Huancheng Road, Jiaxing 314000, Zhejiang, China; ^3^Department of General Surgery, The First Affiliated Hospital of Soochow University, Suzhou, Jiangsu 215004, China; ^4^Department of General Surgery, The First People's Hospital of Yancheng, Yancheng, Jiangsu 224001, China

## Abstract

Worldwide, breast cancer is the most common cancer in females. Endocrine therapy can effectively treat 85% of breast cancer patients, but 15% of patients could only be treated with chemotherapy and surgery, and the prognosis is much worse. Immunotherapy is the novel treatment for breast cancer, where PD-1 and CTLA-4 antibodies have shown evidence of immune modulation in breast cancer drug trials. In this study, we report that TNFRSF9 regulates the cell proliferation, invasion, and apoptosis of breast cancer cells through regulating the phosphorylation of p38, thus further regulating the expression of PAX6. In both breast cancer tissues and cell lines, the levels of TNFRSF9 are significantly decreased, and breast cancer cell development will be promoted with knockdown of TNFRSF9. Moreover, we identify that downregulation of TNFRSF9 can upregulate the phosphorylated p38 (p-p38) and PAX6. We further elucidate that p-p38 is essential for PAX6 expression as p38 phosphorylation inhibitor can reverse the upregulation of PAX6 and suppress cell proliferation and invasion and promote apoptosis in breast cancer cells. In summary, this study proposed a novel TNFRSF9/p38/PAX6 axis that contributes to tumor suppression, which suggests a potential immunotherapy target for breast cancer.

## 1. Introduction

Breast cancer was one of the most common malignant tumors in female. According to the American Cancer Society, in 2017, 255180 new breast cancer cases and 41070 breast cancer deaths are projected to occur in the United States [1]. In China, the incidence and mortality of breast cancer are increasing; in fact, there are 272400 new breast cancer cases and 70700 breast cancer deaths projected to occur in 2015 [2]. Due to highly heterogeneous, potentially aggressive, and complex biological features of breast cancer, more detailed molecular understanding of breast cancer is essential to help develop early diagnosis methods and novel therapies for breast cancer patients.

When the system fails to remove transformed cells, the host develops cancer. Therefore, cancer immunotherapy is to restore or induce immune cells, such as cytotoxic T cells, to identify, target, and destroy cancer cells [3]. The programmed cell death protein 1 (PD-1) mediates the immune checkpoint pathway, and it is normally expressed by activated T cells, B cells, dendritic cells, and natural killer cells. While PD-1 interacts with its ligands, programmed death ligand 1 (PD-L1) and PD-L2, the T cell activation and proliferation are inhibited, preventing autoimmunity in normal tissues [4]. However, several types of tumor cells, including gastrointestinal cancer, ovarian cancer, and breast cancer, are also found to highly express PD-L1, helping the tumor cells escape the immune system. [5–7]. It seems that disrupting the interaction between PD-1 and PD-L1 may promote tumor cells to be attacked by cytotoxic T cells, and after many pieces of research, several PD-1/PD-L1 inhibitors have been developed and showed excellent results in treatment [8,9].

The tumor necrosis factor receptor (TNFR) superfamily member TNFRSF9 (also known as 4-1BB or CD137) is a T cell costimulatory receptor first found to express on activated antigen-presenting cells. Later research also found it on regulatory T cells, activated natural killer T cells, and activated NK cells. [10] The complete roles of TNFRSF9 are not fully activated. TNFRSF9 will induce survival signaling in T cells, but TNFRSF9 deficient T cells are hyperproliferative [11]. Despite the uncertainty, TNFRSF9 has been found to affect tumor cells. Expression of TNFRSF9 on Hodgkin and Reed–Sternberg cells mediates immune escape [12]. It has been reported that TNFRSF9 may play an important role in the mechanism of action of PADI4 related to ovarian tumorigenesis [13]. Many researches show that anti-TNFRSF9 antibodies can boost cancer immunotherapies. For example, the agonistic antibody potentiated the antitumor activity of the anti-HER2 antibody in the mouse models of breast cancer [14]. However, the underlying mechanism is unknown and waiting for exploration.

p38 mitogen-activated protein kinase (p38 MAPK) is an important family member of the MAPK family, and it involves many signal pathways that can affect cell differentiation, apoptosis, and autophagy [15]. Cannons et al. [16] reported that aggregation of TNFRSF9 could induce p38 activation. The transcription factor Paired box 6 (PAX6) is the downstream target of p38, and the transactivation domain of PAX6 will be phosphorylated by p38 [17]. In human cancer, PAX6 is proposed to promote cell proliferation and invasion in colon cancer [18]. The inhibition of PAX6 in lung cancer cell lines also inhibits cell proliferation [19]. Moreover, the overexpression of PAX6 is found in retinoblastoma [20]. In the lymph node metastasis status of breast cancer, PAX6 is switched from methylated to methylation status in the progression of metastasis, which suggests novel biomarkers for breast cancer metastasis [21].

In this study, we reveal that, through upregulating TNFRSF9 in breast cancer, the PAX6 expression can be downregulated through inhibiting p38 phosphorylation and preventing tumor growth. As a result, the agonistic TNFRSF9 antibody may be a novel molecule for breast cancer therapies.

## 2. Methods

### 2.1. Patient Samples

A total of 30 pairs of breast cancer tissues and paracancerous tissues were collected from breast cancer patients from the Second Affiliated Hospital of Jiaxing University. All the patients were female and aged between 30 and 60 years. They were pathologically diagnosed as nonspecific invasive breast cancer for the first time and had not received chemotherapy before the study. Clinicopathological features of patients were collected for further data analysis. This study was approved by the Ethics Committee of the Second Affiliated Hospital of Jiaxing University (JXEY-ZFYJ041) and the Laboratory Animal Ethics Committee of JXMC (JUMC2020-131). All related patients were informed about the research and have signed the informed consent letters. After extraction, the tissues were quickly frozen and stored at −80°C for RNA and protein extraction.

### 2.2. Cell Lines and Cell Culture

The breast cancer cell lines, including MCF-7, MDA-MB-453, MDA-MB-231, ZR-75-30, BT474, and human nontumorigenic breast epithelial cell line MCF-10A, were cultured in 10% FBS DMEM medium under an environment of 5% CO_2_ and 37°C.

### 2.3. Cell Transfection

Small interfering RNA against TNFRSF9 (si-TNFRSF9) and small interfering RNA negative control (si-NC) were used to knock down the expression of TNFRSF9 in the MCF-7 and ZR-75-30 cell lines. pcDNA 3.1 plasmids with the full length of TNFRSF9 (pcDNA3.1-TNFRSF9) and related negative control (NC) were used to upregulate TNFRSF9 in the MCF-7 and ZR-75-30 cell lines. All mentioned factors were then transfected into cells through Lipofectamine™ 2000, adhered to the provided protocol.

### 2.4. Quantitative Real-Time PCR (qRT-PCR)

Total RNA in breast cancer tissues, paracancerous tissues, and cells was extracted by TRIzol reagent. qRT-PCR was performed by using the ABI 7900HT RealTime PCR System based on SYBR Green assay. The 2^−ΔΔCt^ method was used to evaluate the RNA expression level.

### 2.5. Western Blot

The whole-cell lysate was prepared in RIPA protein solubilization buffer and membrane Protein Extraction Kit (Solarbio Life Science; EX1110) was used to extract TNFRSF9 protein. Proteins were quantified using BCA kits and blocked with 5% skim milk at room temperature for one hour. After washing with PBS three times, proteins were incubated with primary antibodies (TNFRSF9, p-p38 MAPK, p38 MAPK, PAX6, and GAPDH) overnight at 4°C. The membrane was then washed with PBS 3 times, 5 minutes each, followed with incubation with secondary antibody (1 : 2000) and incubation at 25°C for 2 hours. The band was visualized using ECL luminescence solution in a dark room. The images were developed and analyzed with the ChemiDocXRS + system.

### 2.6. Cell Proliferation Detection

Cell Counting Kit-8 (CCK-8) was used to detect cell proliferation. Cells of each group (5 × 105 cells/ml) were incubated in a 96-well plate (100 *μ*l/well) for 72 hours in an incubator. After removing the medium, 10 *μ*l CCK-8 solution was added to each well of the plate. The plate was incubated in the dark for 3 hours. The results were determined by measuring the light absorbance at 450 nm on a microplate reader.

5′-Bromo-2′-deoxyuridine (BrdU) assay was also performed to examine the cell proliferation ability of the MCF-7 cells and ZR-75-30 cells. 10 *μ*M of BrdU was added into the investigated cells and incubated for 1 hour. After that, the cells were resuspended at 4°C and frozen in 70% V/V ethanol. The cells were collected and washed with PBS by centrifuge. Then the cells were cultured with HCl (2 M) at room temperature for a half hour. After washing twice with PBS, the anti-BrdU antibody was applied to the cells and cultured in the dark for 20 minutes. Finally, images were developed with fluorescent microscopy.

### 2.7. Cell Invasion

Transwell assay was used to assess the cell invasion ability of MCF-7 cells and ZR-75-30 cells. The top chamber of Transwell was pretreated with 300 *μ*l serum-free media and incubated for 2 hours at room temperature. Each group of cells were suspended in serum-free media and diluted to 5 × 10^5^ cell/ml. 300 *μ*l cell suspension was added into the top chamber of Transwell, and the lower chamber was filled with 500 *μ*l medium containing FBS. After 24 hours of incubation at 37°C, the inserts were stained with 0.1% crystal violet for another 20 minutes. After washing, five views (200x) of the upper chamber's lower layer were chosen randomly to observe and count the invaded cells under the microscope.

### 2.8. Cell Apoptosis Evaluation

Cell apoptosis was detected through Annexin V-FITC/PI double staining and was analyzed by flow cytometry. Cells were harvested and washed with PBS and then resuspended in 500 *μ*l binding buffer, which contained 5 *μ*L Annexin V-FITC and 5 *μ*L propidium iodide. After 10 minutes of incubation in the dark at room temperature, the samples were applied for flow cytometry (FCM) analysis.

### 2.9. Xenograft Subcutaneous Tumor Formation Assay

Five to six weeks' old female BALB/C mice were anesthetized and were given a dorsal subcutaneous injection of MCF-7 cells, suspended in 100 *μ*l of Matrigel (*n* = 10). Five mice's tumors were injected with the pLV-TNFRSF9 plasmid, and five tumors were injected with the negative control plasmid (NC). The volume of the tumor was measured every three days after the therapy. The mice were euthanized after 18 days, and the areas containing transplanted cells were measured, sliced, and stained with H&E, Bcl-2 antibodies, or TNFRSF9 antibodies. Animal studies were approved by the ethics committee of the Second Affiliated Hospital of Jiaxing University and followed the guidelines of Care and Use of Laboratory Animals of China.

### 2.10. Statistical Analysis

Statistical analysis was performed using SPSS software (version 22.0). Data were presented as mean ± standard deviation (*x* ± *s*) of at least three biological repeats. *t*-test was used for the intergroup comparison, and two-sided chi-squared test was used to compare groups. Values of *p* < 0.05 indicate statistical significance.

## 3. Results

The expression of TNFRSF9 in breast cancer is significantly decreased and related to metastasis and malignant.

**Figure 1:**
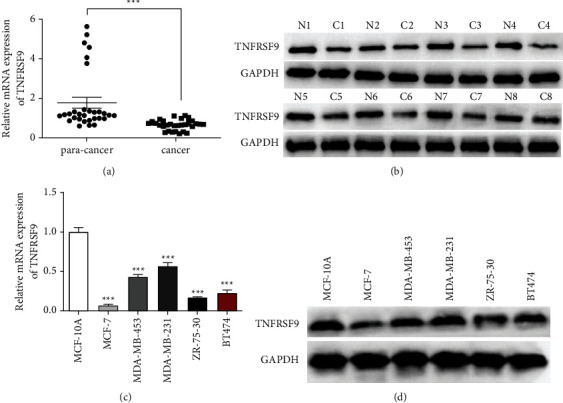
The expression of TNFRSF9 in breast cancer decreased. (a) qRT-PCR assay for TNFRSF9 mRNA expression in breast cancer tissues (*n* = 10) and paracancerous tissues. (b) Western blot assays for TNFRSF9 protein expression in breast cancer tissues (*n* = 8) and paracancerous tissues. (c) qRT-PCR assay for TNFRSF9 mRNA expression in breast cancer cell lines. (d) Western blot assays for TNFRSF9 protein expression in breast cancer cell lines. ^*∗∗∗*^*p* < 0.001.

We conducted qRT-PCR assays to detect the mRNA expression of TNFRSF9 in breast cancer tissues and paracancerous normal tissues collected from 30 patients. As shown in Figure 1(a), the expression of TNFRSF9 in breast cancer tissues was remarkably lower than their nearby nontumor tissues (^*∗∗∗*^*p* < 0.001). The western blot assay result (Figure 1(b)) indicates the TNFRSF9 protein expression's downregulation in eight matched breast cancer tissues and normal tissues. Low expression of TNFRSF9 mRNA and TNFRSF9 protein was observed in five breast cancer cell lines (Figures 1(c)-1(d), ^*∗∗∗*^*p* < 0.001). We also compared the clinicopathological features between low TNFRSF9 expression patients and high TNFRSF9 expression patients (Table 1). Patients with high TNFRSF9 were less likely to have lymphoid metastasis (*p*=0.0007) and lower malignant tumors (TNM) stage (*p*=0.0016). The above results indicate that dysregulation of TNFRSF9 may have an essential role in breast cancer progression.

### 3.1. Regulating TNFRSF9 Expression Can Mediate p38 Phosphorylation and Further Affect the Expression of PAX6

**Figure 2:**
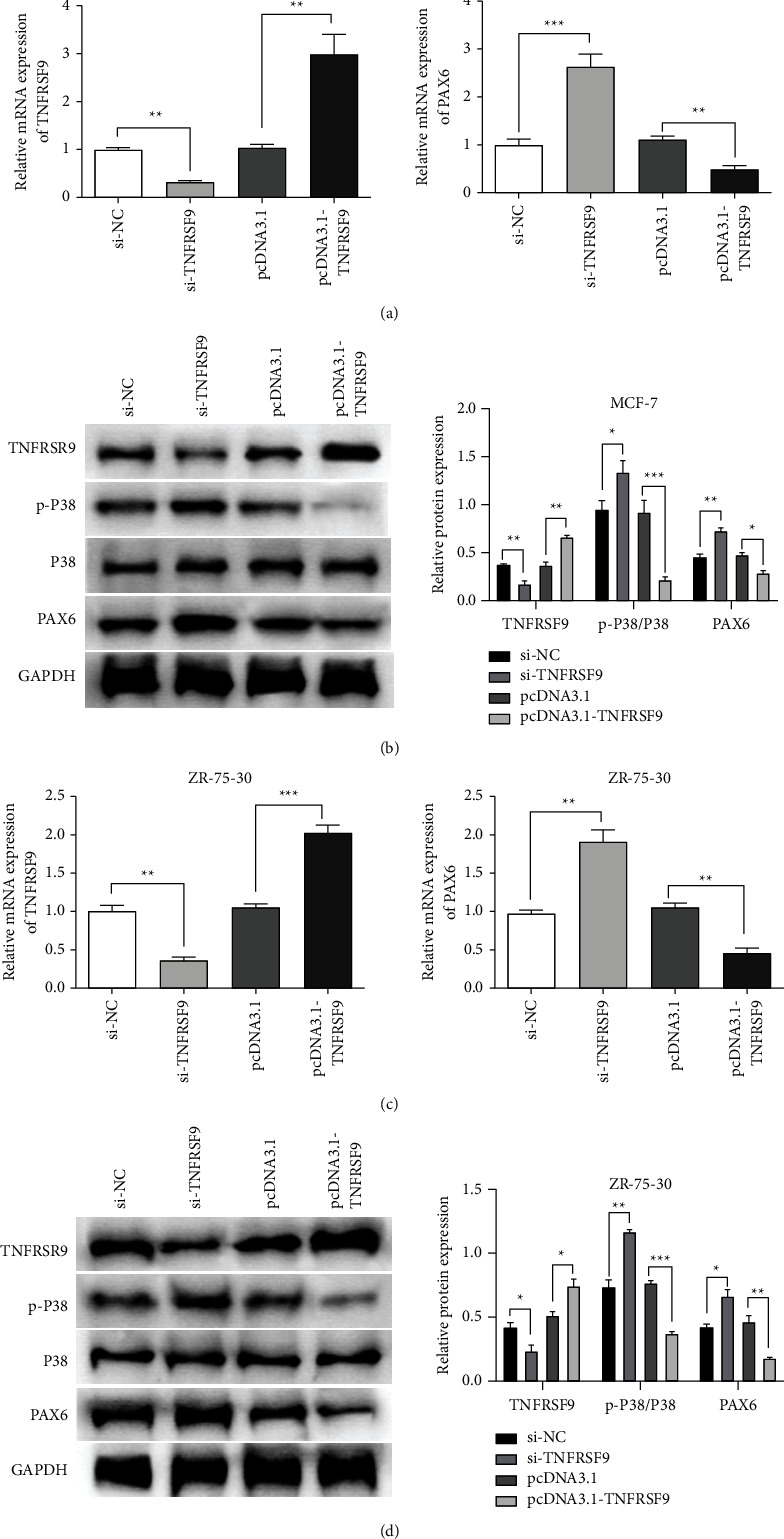
TNFRSR9 mediated p38 phosphorylation and PAX6 expression. (a, c) qRT-PCR assays for TNFRSF9 mRNA expression (left panel) and PAX6 mRNA expression (right panel) in MGF-7 cells (a) or ZR-75-30 cells (c) transfected with si-TNFRSF9 or pcDNA3.1-TNFRSF9. (b, d) Western blot assays for TNFRSF9, p-P38, P38, and PAX6 protein expression (left panel) and quantification of protein expression (right panel) in MGF-7 cells (b) or ZR-75-30 cells (d) transfected with si-TNFRSF9 or pcDNA3.1-TNFRSF9.

To further investigate the role of TNFRSF9 in breast cancer, we knocked down TNFRSF9 through transfecting MCF-7 cells and ZR-75-30 with si-TNFRSF9, as these two cell lines have the lowest TNFRSF9 expression in all the breast cancer cell lines. We also overexpressed TNFRSF9 by transfecting MCF-7 cells and ZR-75-30 with pcDNA3.1-TNFRSF9. qRT-PCR assays were used to determine expression regulation efficiency (Figure 2(a) left panel and Figure 2(c) left panel, ^*∗∗*^*p* < 0.01, ^*∗∗∗*^*p* < 0.001).

We first confirmed the relationship between TNFRSF9, p38, and PAX6. The mRNA expression of PAX6 was detected using qRT-PCR assays. In both the MCF-7 cell line (Figure 2(a) right panel) and ZR-75-30 cell line (Figure 2(c) right panel), the mRNA expression of PAX6 was increased with TNFRSF9 downregulation (^*∗∗*^*p* < 0.01, ^*∗∗∗*^*p* < 0.001) and decreased with TNFRSF9 upregulation (^*∗∗*^*p* < 0.01). As the phosphorylation status of p38 cannot be determined by mRNA, western blot assays were used to assess p-p38 protein expression. In both the MCF-7 cell line (Figure 2(b)) and ZR-75-30 cell line (Figure 2(d)), with TNFRSF9 knockdown, TNFRSR9 protein expression decreased, p-p38 protein expression increased, and PAX6 protein expression increased. In contrast, p38 protein expression remained not significantly changed compared with the knockdown negative controls (^*∗*^*p* < 0.05, ^*∗∗*^*p* < 0.01). Furthermore, we also added the expression of TNFRSF9 by flow cytometry analysis; the result is provided in Supplementary Figure 1. In contrast, with TNFRSF9 overexpression, TNFRSR9 protein expression increased, p-P38 protein expression decreased remarkably, and PAX6 protein expression decreased while p38 protein expression remained not significantly changed when compared with the overexpression negative controls (^*∗*^*p* < 0.05, ^*∗∗*^*p* < 0.01, ^*∗∗∗*^*p* < 0.001). The above results indicate that TNFRSR9 mediates p38 phosphorylation and PAX6 protein expression.

### 3.2. Dysregulated TNFRSF9 Expression Affected Cell Proliferation, Invasion, and Apoptosis

**Figure 3:**
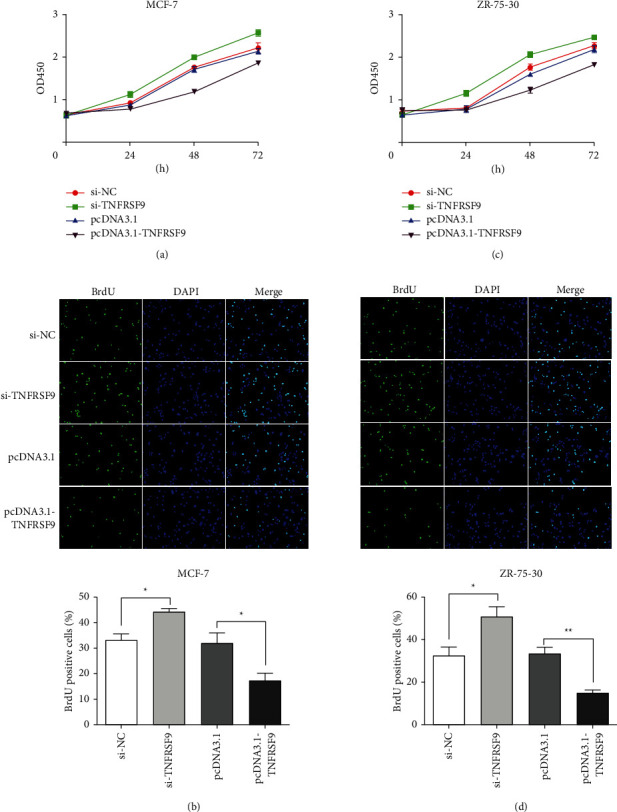
TNFRSF9 expression affected cell proliferation. (a, b) Cell proliferation assay performed in MGF-7 cells transfected with si-TNFRSF9 or pcDNA3.1-TNFRSF9, including CCK-8 assay (a) and BrdU assay (b). (c, d) Cell proliferation assay performed in ZR-75-30 cells transfected with si-TNFRSF9 or pcDNA3.1-TNFRSF9, including CCK-8 assay (c) and BrdU assay (d). ^*∗*^*p* < 0.05; ^*∗∗*^*p* < 0.01.

**Figure 4:**
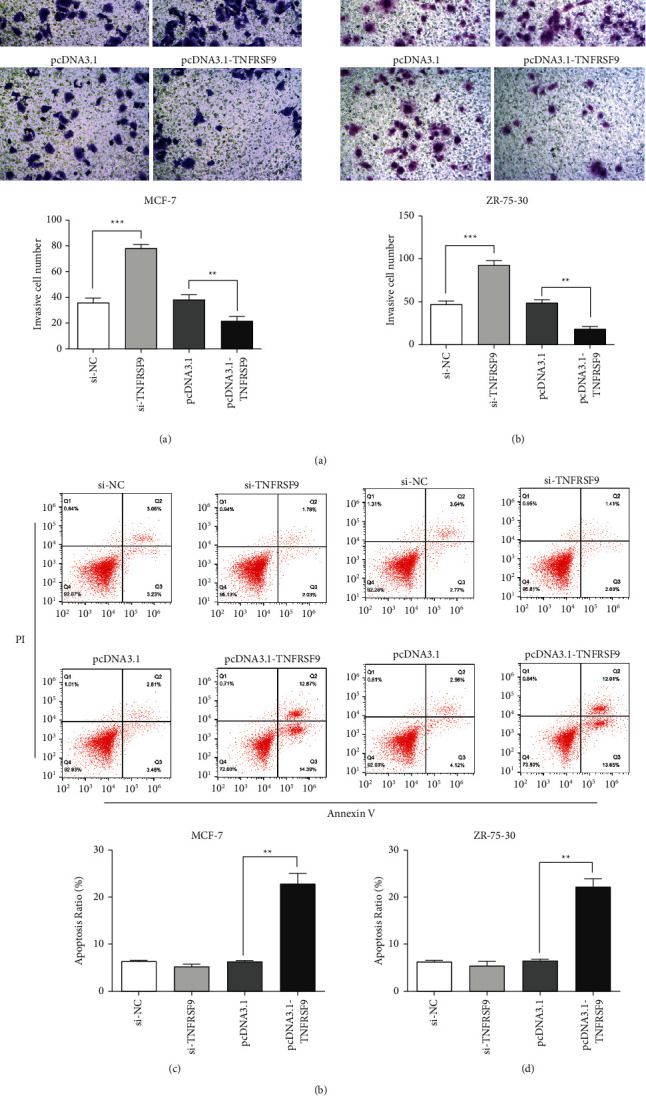
TNFRSF9 expression affected cell invasion ability and apoptosis. (a, b) The invasion ability of MGF-7 cells (a) or ZR-75-30 cells (b) transfected with si-TNFRSF9 or pcDNA3.1-TNFRSF9 was determined using Transwell invasion assay. Quantitative analysis of the invasive cells in Transwell assays was also performed (lower panel). (c-d) Apoptosis assays of MGF-7 cells (c) or ZR-75-30 cells (d) transfected with si-TNFRSF9 or pcDNA3.1-TNFRSF9, and the quantitative analysis of the apoptosis cells (lower panel). ^*∗∗*^*p* < 0.01; ^*∗∗∗*^*p* < 0.001.

Next, we investigated the dysregulation effect of TNFRSF9 in tumor development. The CCK-8 assays were performed to exam the cell proliferation. In both the MCF-7 cell line (Figure 3(a)) and ZR-75-30 cell line (Figure 3(c)), when compared with the negative controls, cell proliferation increased with TNFRSF9 knockdown and decreased with TNFRSF9 overexpression. BrdU assays were also performed to exam the cell proliferation. Similarly, comparing with the negative controls, in both MCF-7 cell line (Figure 3(b)) and ZR-75-30 cell line (Figure 3(d)), cell proliferation increased with TNFRSF9 knockdown (^*∗*^*p* < 0.05) and decreased with TNFRSF9 overexpression (^*∗*^*p* < 0.05, ^*∗∗*^*p* < 0.01). Transwell assays were performed to investigate the cell invasion ability (Figures 4(a) and 4(b)). Comparing with the negative control, in both cell lines, knockdown TNFRSF9 enhanced cell invasion ability (^*∗∗∗*^*p* < 0.001) and overexpress TNFRSF9 impaired cell invasion ability (^*∗∗*^*p* < 0.01). The cell apoptosis was examined through flow cytometry in both cell lines (Figures 4(c) and 4(d)). The apoptotic rate decreased increased when overexpressed TNFRSF9 (^*∗∗*^*p* < 0.01). These results suggest that TNFRSF9 is a suppresser of breast cancer malignancy and activating TNFRSF9 could suppress breast cancer progression.

TNFRSF9 mediated PAX6 through p-p38, and inhibiting p38 phosphorylation could restore PAX6 expression from TNFRSF9 knockdown.

**Figure 5:**
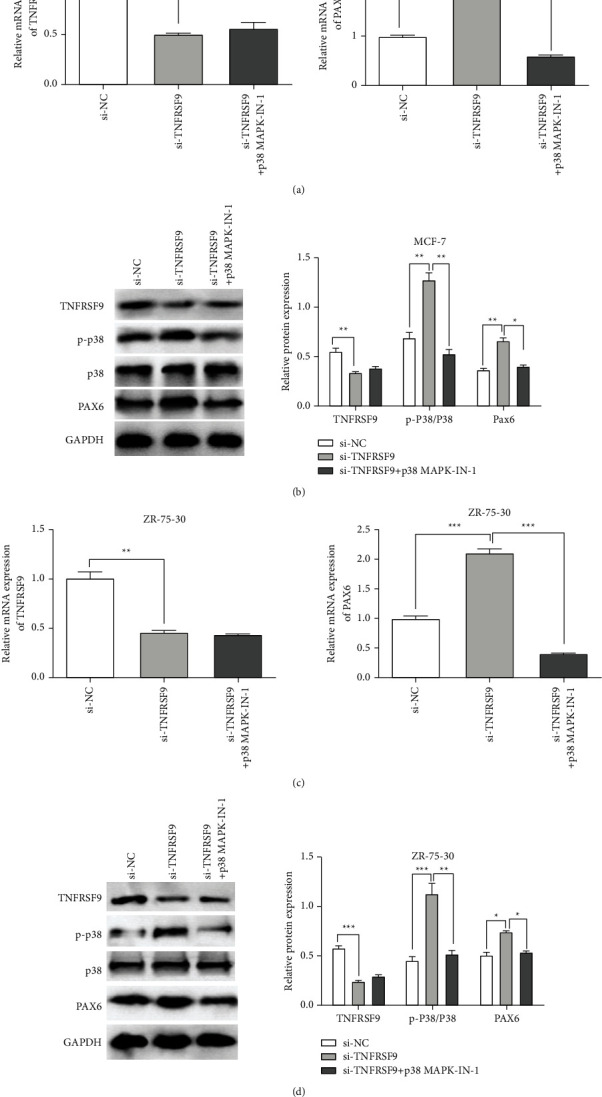
Inhibition of p38 phosphorylation reversed PAX6 upregulation caused by TNFRSF9 knockdown. (a, c) qRT-PCR assays for TNFRSF9 mRNA expression (left panel) and PAX6 mRNA expression (right panel) in MGF-7 cells (a) or ZR-75-30 cells (c) transfected with si-TNFRSF9 and with or without p38 MAPK-IN-1. (b, d) Western blot assays for TNFRSF9, p-P38, P38, and PAX6 protein expression (left panel) and quantification of protein expression (right panel) in MGF-7 cells (b) or ZR-75-30 cells (d) transfected with si-TNFRSF9 and with or without p38 MAPK-IN-1. ^*∗*^*p* < 0.05, ^*∗∗*^*p* < 0.01, and ^*∗∗∗*^*p* < 0.001.

We found that TNFRSF9 dysregulation affects p38 phosphorylation but not p38 expression previously, so we inhibited p38 phosphorylation to determine the regulation axis. qRT-PCR assays and western blot assays were used to determine the efficiency of the p38 phosphorylation inhibitor (p38 MAPK-IN-1). In both the MCF-7 cell line (Figure 5(a)) and ZR-75-30 cell line (Figure 5(c)), when knocking down TNFRSF9 with p38 phosphorylation inhibitor, the downregulated TNFRSF9 mRNA expression was not affected, and the mRNA expression of PAX6 was decreased compared to the negative control level (^*∗∗*^*p* < 0.01, ^*∗∗∗*^*p* < 0.001). The western blot assays (Figure 5(b) and Figure 5(d)) confirmed that the p38 phosphorylation inhibitor inhibited the protein expression of p-p38 without affecting TNFRSF9 protein expression and restored the PAX6 protein expression to low level as the negative control (^*∗*^*p* < 0.05, ^*∗∗*^*p* < 0.01, ^*∗∗∗*^*p* < 0.001).

**Figure 6:**
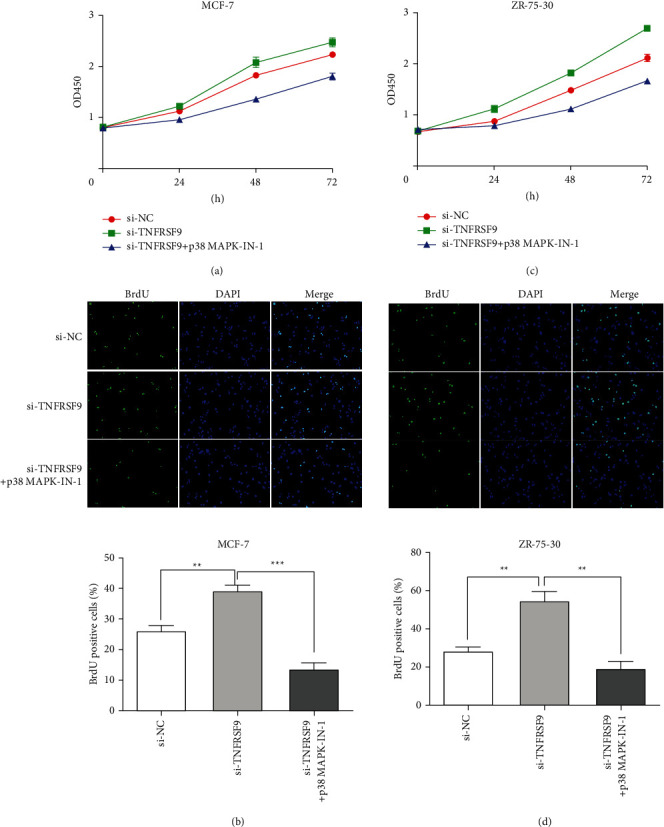
Inhibition of p38 phosphorylation reversed the changes in cell proliferation caused by TNFRSF9 knockdown. (a, b) Cell proliferation assay performed in MGF-7 cells transfected with si-TNFRSF9 and with or without p38 MAPK-IN-1, including CCK-8 assay (a) and BrdU assay (b). (c, d) Cell proliferation assay performed in ZR-75-30 cells transfected with si-TNFRSF9 and with or without p38 MAPK-IN-1, including CCK-8 assay (c) and BrdU assay (d). ^*∗∗*^*p* < 0.01^*∗∗*^*p* < 0.01; ^*∗∗∗*^*p* < 0.001.

**Figure 7:**
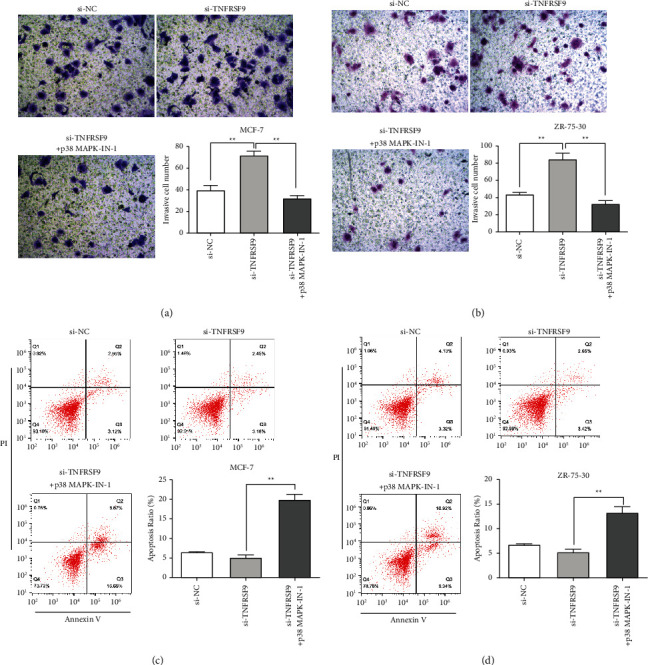
Inhibition of p38 phosphorylation reversed the changes in cell invasion ability and apoptosis caused by TNFRSF9 knockdown. (a, b) The invasion ability of MGF-7 cells (a) or ZR-75-30 cells (b) transfected with si-TNFRSF9 and with or without p38 MAPK-IN-1 was determined using Transwell invasion assay. Quantitative analysis of the invasive cells in Transwell assays was also performed (lower right panel). (c, d) Apoptosis assays of MGF-7 cells (c) or ZR-75-30 cells (d) transfected with si-TNFRSF9 and with or without p38 MAPK-IN-1 and the quantitative analysis of the apoptosis cells (lower right panel). ^*∗∗*^*p* < 0.01.

We further investigated the restoration effect of p38 in tumor development. The CCK-8 assays were performed to examine the cell proliferation. In both the MCF-7 cell line (Figure 6(a)) and ZR-75-30 cell line (Figure 6(c)), comparing with the TNFRSF9 knockdown group, cell proliferation decreased with p38 phosphorylation inhibitor. BrdU assays were also performed to examine the cell proliferation. Similarly, comparing with the TNFRSF9 knockdown group, in both MCF-7 cell line (Figure 6(b)) and ZR-75-30 cell line (Figure 6(d)) cell proliferation decreased significantly with p38 phosphorylation inhibitor (^*∗∗*^*p* < 0.01, ^*∗∗∗*^*p* < 0.001). Transwell assays were performed to investigate the cell invasion ability (Figures 7(a) and 7(b)). Comparing with the TNFRSF9 knockdown group, in both MCF-7 and ZR-75-30 cell lines, inhibiting p38 phosphorylation impaired cell invasion ability compared to the negative control level (^*∗∗*^*p* < 0.01). The cell apoptosis was examined through flow cytometry in both cell lines (Figures 7(c) and 7(d)). Compared with si-TNFRSF9 group, the apoptotic rate increased when p38 MAPK-IN-1 was given to si-TNFRSF9 transfected cells (^*∗∗*^*p* < 0.01). All these findings suggest that TNFRSF9 suppresses breast cell carcinogenesis through inhibiting p38 phosphorylation and inhibits PAX6 protein expression as a result.

### 3.3. Overexpression of TNFRSF9 Can Reduce Malignancy in Breast Cancer Cell-Induced Tumor Formation

**Figure 8:**
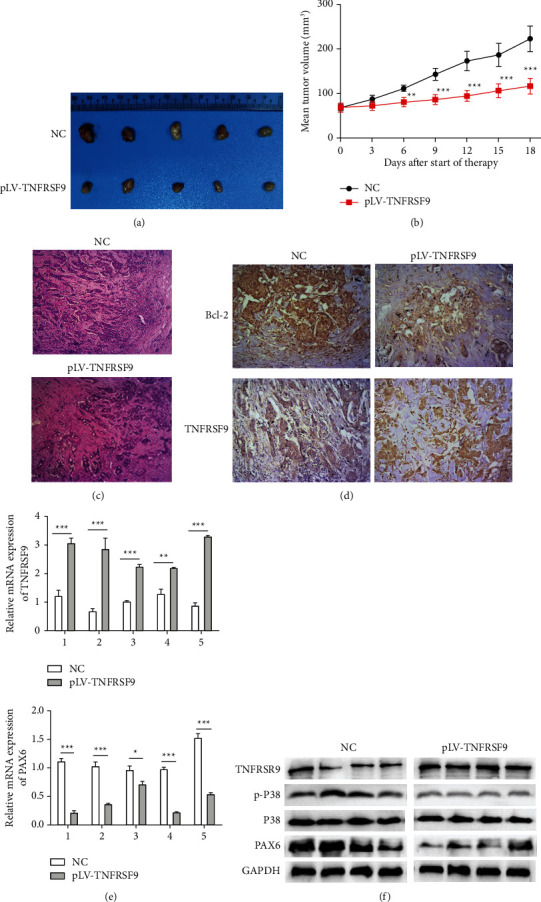
TNFRSF9 overexpression inhibited breast cancer cell-induced tumor formation. (a) The representative appearances of extirpated xenograft tumors with implantation of pLV-TNFRSF9 transfected MCF-7 cells. (b) The size of tumors with implantation of pLV-TNFRSF9 transfected MCF-7 cells. (c) Pathological H&E stained image of tumors with implantation of pLV-TNFRSF9 transfected MCF-7 cells. (d) Expression of Bcl-2 (upper panel) and TNFRSF9 lower panel) in tumors with implantation of pLV-TNFRSF9 transfected MCF-7 cells were detected by immunohistochemistry. (e) qRT-PCR assays for TNFRSF9 mRNA expression (upper panel) and PAX6 mRNA expression (lower panel) in tumors with implantation of pLV-TNFRSF9 transfected MCF-7 cells. (f) Western blot assays for TNFRSF9, p-P38, P38, and PAX6 protein expression in tumors with implantation of pLV-TNFRSF9 transfected MCF-7 cells. ^*∗*^*p* < 0.05, ^*∗∗*^*p* < 0.01, and ^*∗∗∗*^*p* < 0.001.

We further gave a subcutaneous injection of MCF-7 cells transfected with or without pLV-TNFRSF9 to mice to induce tumor formation. Tumors induced by pLV-TNFRSF9 injected MCF-7 cells were significantly smaller in size compared to tumors induced by NC injected MCF-7 cells (Figures 8(a) and 8(b)). Furthermore, the tumors' pathological slides were stained with H&E (Figure 8(c)), Bcl-2 antibody, or TNFRSF9 antibody (Figure 8(d)). The H&E image indicated that the pLV-TNFRSF9 injected tumor was less malignant. The immunohistochemical image showed less Bcl-2 expression and more TNFRSF9 expression in the pLV-TNFRSF9 injected tumor. qRT-PCR assays were performed to evaluate the mRNA expression of TNFRSF9 and PAX6 in the tumor tissues (Figure 8(e)). In all five pLV-TNFRSF9 injected tumors, the mRNA expression of TNFRSF9 was significantly upregulated (upper panel) and the mRNA expression of PAX6 was significantly downregulated (lower panel) (^*∗*^*p* < 0.05, ^*∗∗*^*p* < 0.01, ^*∗∗∗*^*p* < 0.001). The western blot assay result (Figure 8(f)) indicates that, in eight matched tumor tissues, the protein expression of TNFRSF9 was significantly promoted with pLV-TNFRSF9 injection. In contrast, the protein expression of p-p38 and PAX6 was significantly suppressed, and the protein expression of p38 remains unchanged. Therefore, agonistic TNFRSF9 antibody may be a novel therapy for breast cancer patients.

## 4. Discussion

Breast cancer is the most common cancer in women all around the world. Although the endocrine therapy is potent that the five-year breast cancer-specific survival could be over 99%, only 70% of breast cancer patients whose tumor cells overexpressed estrogen receptor or progesterone receptor proteins fit this therapy. For the 15% triple-negative breast cancer (TNBC) patients whose tumor does not overexpress estrogen receptor, progesterone receptor, or HER2, they can only be treated with chemotherapy and surgery, have a worse prognosis, and are more likely to relapse [22].

Immunotherapy is a revolutionized cancer therapy that activates the tumor-specific immune response to eradicate the tumor. Several immune biomarkers have been identified in breast cancer, which can be used for prognosis and prediction. For example, HER-2^+^ breast cancers and TNBCs are more likely to express the PD-L1 in the tumor microenvironment than luminal breast cancers [23,24]. Therefore, breast cancer is very suitable for immunotherapy, and several drugs have been tested in breast cancer trials with evidence of immune modulation. CTLA-4 expresses T cell and binding CD80/CD86 to limiting T cell activation during the immune response's priming phase. Two anti-CTLA-4 agents, tremelimumab and ipilimumab, have been developed and used in many tumor types. In a breast cancer drug test, tremelimumab significantly increased the ratio of ICOS^+^/FoxP3^+^ CD4^+^ T cells in most patients and stablized the disease for more than 12 weeks in 42% of patients [25]. Several agonistic antibodies targeting other inhibitory immune checkpoint molecules such as lymphocyte-activation gene 3 (LAG3) and T cell immunoglobulin and mucin domain-containing molecule 3 (TIM3) are also in development [26]. In our study, we reveal a possible inhibitory immune checkpoint molecule TNFRSF9 for breast cancer treatment where the agonistic antibody of TNFRSF9 may be effective for breast cancer immunotherapy.

We show that TNFRSF9 inhibits tumor progression by suppressing p38 phosphorylation and the protein expression of the downstream target of p-p38 and PAX6. PAX genes are essential for cancer cell survival [27]. According to Zong et al. [28], downregulation of PAX6 in vitro can suppress cell viability, DNA synthesis, and colony formation. The tumorigenesis in xenograft nude mice was also significantly inhibited. In this study, we confirmed that downregulation of PAX6 expression could suppress breast cancer development in vivo and inhibit tumorigenesis in xenograft nude mice. We also revealed that TNFRSF9 and p-p38 were the upstream regulators of PAX6. 33, we did not confirm that p38 is the direct downstream target of TNFRSF9 or PAX6 is the direct target of p-p38. Jie et al. have reported that *β*-catenin binds to the proximal region of PAX6 promoter during osteoclast differentiation to induce PAX6 expression, and p38 could cause *β*-catenin degradation. Thus, there is a p38/ *β*-catenin/PAX6 axis in the negative regulation of osteoclastogenesis [29]. It is highly likely that a similar axis exists in breast cancer cells between TNFRSF9 and p38 or p38 and PAX6, which could be further investigated in the future.

## 5. Conclusion

In conclusion, we disclosed for the first time that PAX6 is regulated by a novel TNFRSF9/p38 pathway. PAX6 can further promote breast cancer development. Our study indicates that this novel TNFRSF9/p38/PAX6 axis contributes to suppressing tumorigenesis. We also propose an agonistic TNFRSF9 antibody as a potential antibody for breast cancer immunotherapy [31].

## Figures and Tables

**Table 1:** Association analysis of TNFRSF9 expression and the clinicopathological features in 30 breast cancer patients.

Characteristics	Case number	TNFRSF9 expression		*p* value
		Low (*n* = 20)	High (*n* = 10)	
Number	30	20	10	
Ages (years)				0.5921^a^
≤55	19	12	7	
>55	11	8	3	
Tumor size				0.5839^a^
≤2 cm	10	6	4	
>2 cm	20	14	6	
HR (estrogen receptor) status				0.6048^a^
Negative	14	10	4	
Positive	16	10	6	
HER2 status				0.7945 ^a^
Negative	17	11	6	
Positive	13	9	4	
Lymph node metastasis				0.0007 ^a^
No	9	2	7	
Yes	21	18	3	
TNM stage				0.0016^a^
I–II	12	4	8	
III	18	16	2	

^a^Two-sided chi-squared test.

## Data Availability

The datasets analyzed for the present study are available from the corresponding author upon reasonable request.

## Supplementary Materials

Supplementary Materials Supplementary Figure 1: the expression of TNFRSF9 in MCF-7 cells was confirmed by flow cytometry analysis.
