# Supplementary figures and images for: TNFRSF9 Suppressed the Progression of Breast Cancer via the p38MAPK/PAX6 Signaling Pathway
Source: J Oncol. 2022 Jun 28;2022:8549781. doi: 10.1155/2022/8549781 (PMC9256432; doi:10.1155/2022/8549781)

Supplementary Figure 1

si-NC

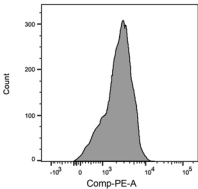

si-TNFRSF9

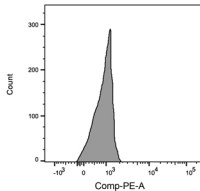

pcDNA3.1-TNFRSF9

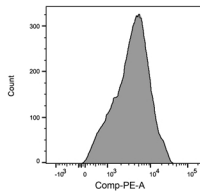

si-TNFRSF9+p38 MAPK-IN-1

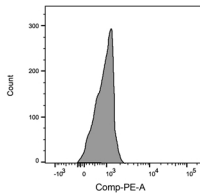

Supplement: Supplementary Materials — Supplementary Figure 1: the expression of TNFRSF9 in MCF-7 cells was confirmed by flow cytometry analysis. [file 8549781.f1.pdf]
